# CaMKII activation in early diabetic hearts induces altered sarcoplasmic reticulum-mitochondria signaling

**DOI:** 10.1038/s41598-021-99118-x

**Published:** 2021-10-08

**Authors:** Marilen Federico, Maite Zavala, Tamara Vico, Sofía López, Enrique Portiansky, Silvia Alvarez, Maria Celeste Villa Abrille, Julieta Palomeque

**Affiliations:** 1 Centro de Investigaciones Cardiovasculares, UNLP-CONICET-CCT La Plata, Facultad de Ciencias Médicas, UNLP, 60 y 120 s/n, La Plata, CP 1900 Argentina; 2 Instituto de Bioquímica y Medicina Molecular, UBA-CONICET, Facultad de Farmacia y Bioquímica, Buenos Aires, Argentina; 3 Laboratorio de Análisis de Imágenes, UNLP, Facultad de Ciencias Veterinarias, La Plata, Argentina

**Keywords:** Physiology, Cardiovascular biology

## Abstract

Prediabetic myocardium, induced by fructose-rich diet (FRD), is prone to increased sarcoplasmic reticulum (SR)-Ca^2+^ leak and arrhythmias due to increased activity of the Ca^2+^/calmodulin protein kinase II (CaMKII). However, little is known about the role of SR-mitochondria microdomains, mitochondrial structure, and mitochondrial metabolisms. To address this knowledge gap we measured SR-mitochondrial proximity, intracellular Ca^2+^, and mitochondrial metabolism in wild type (WT) and AC3-I transgenic mice, with myocardial-targeted CaMKII inhibition, fed with control diet (CD) or with FRD. Confocal images showed significantly increased spontaneous Ca^2+^ release events in FRD vs. CD WT cardiomyocytes. [^3^H]-Ryanodine binding assay revealed higher [^3^H]Ry binding in FRD than CD WT hearts. O_2_ consumption at State 4 and hydrogen peroxide (H_2_O_2_) production rate were increased, while respiratory control rate (RCR) and Ca^2+^ retention capacity (CRC) were decreased in FRD vs. CD WT isolated mitochondria. Transmission Electron Microscopy (TEM) images showed increased proximity at the SR-mitochondria microdomains, associated with increased tethering proteins, Mfn2, Grp75, and VDAC in FRD vs. CD WT. Mitochondria diameter was decrease and roundness and density were increased in FRD vs. CD WT specimens. The fission protein, Drp1 was significantly increased while the fusion protein, Opa1 was unchanged in FRD vs. CD WT hearts. These differences were prevented in AC3-I mice. We conclude that SR-mitochondria microdomains are subject to CaMKII-dependent remodeling, involving SR-Ca^2+^ leak and mitochondria fission, in prediabetic mice induced by FRD. We speculate that CaMKII hyperactivity induces SR-Ca^2+^ leak by RyR2 activation which in turn increases mitochondria Ca^2+^ content due to the enhanced SR-mitochondria tethering, decreasing CRC.

## Introduction

In adult cardiomyocytes, excitation-contraction-bioenergetics coupling (ECB) is the process that links electrical stimulation, at the cell surface, with myofilament interaction, that drives contraction and with mitochondria energetics, that fulfills cardiac ATP demands. In each one of these processes, calcium ions (Ca^2+^) are primarily involved. When cardiomyocytes are stimulated by an action potential, depolarization of the plasma membrane allows Ca^2+^ entry through L-type Ca^2+^ channels (LTCC). Ca^2+^ binds to ryanodine receptors (RyR2) at the sarcoplasmic reticulum (SR) to produce Ca^2+^-induced-Ca^2+^-release (CICR)^1^, which subsequently activates myofilaments for muscle contraction. Relaxation occurs when the SR Ca^2+^ ATPase (SERCA2a) reuptakes Ca^2+^, lowering cytosolic Ca^2+^ concentration in combination with Ca^2+^ extrusion via the Na^+^-Ca^2+^ exchanger (NCX)^2^. The sarcolemma Ca^2+^-ATPase and mitochondrial calcium uniporter (MCU) also participate in decreasing systolic Ca^2+^ concentration, although to a much lesser extent than the previously mentioned transporters^3^. Mitochondrial Ca^2+^^4^ is essential for ATP and reactive oxygen species (ROS) production/elimination, warranting cell functioning, and survival^5^. Indeed, mitochondrial Ca^2+^ is one of the major activators of Ca^2+^-dependent dehydrogenases in the tricarboxylic acid (TCA) cycle, which in turn can produce an increase in ATP or reactive oxygen species (ROS) production^6,7^. However, mitochondria can also determine the progression of cell death with great relevance in cardiovascular (patho)physiology. In this scenario, Ca^2+^ also plays a pivotal role in opening mitochondria permeability transition pore (mPTP) when reaching a threshold level, enabling the release of ions and solutes from the mitochondrial matrix and thereby triggering programmed cell death^8^.

It is well known that mitochondria are dynamic organelles, which constantly change their size, location, and shape, by fusion and fission. This two complementary processes^9^, are mainly carried out by different proteins. In the case of fusion, GTPases such as mitofusin 1 and 2 (Mfn1/2), located in the outer mitochondrial membrane (OMM), and optic protein atrophy 1 (Opa1), in the inner mitochondrial membrane (IMM) are major recognized players. On the other hand, dynamin-related protein 1 (Drp1) is the most important protein involved in fission^10,11^. Despite these described canonical roles, these proteins can exert non-canonical functions, i.e. Mfn2 and Drp1 may participate as a tether^12–14^ and Opa1 as a maintainer of cristae structure^15^. Both fission and fusion work in concert to sustain mitochondria physiological function, including mitochondrial DNA stability, respiratory capacity, apoptosis, response to cellular stress, and mitophagy^16^. In this context, the mitochondrial balance between fusion and fission is essential in mammals, and even mild defects in mitochondrial dynamics are associated with diseases. Besides, fission is usually associated with deleterious processes and fusion with compensatory mechanisms, although both processes are necessary during development^17,18^.

In previous studies performed on early diabetic animals induced by fructose-rich diet (FRD), a standardized prediabetic model characterized by impaired glucose tolerance and insulin resistance^19–21^, we found that Ca^2+^ handling was altered, inducing cardiac arrhythmias and apoptosis due to Ca^2+^/calmodulin kinase II (CaMKII) hyperactivity and phosphorylation of RyR2^21,22^. It has been described that in type 2 diabetes mellitus (T2DM) hearts (*db/db* mice), arrhythmia susceptibility is due to cardiac sympathetic dysfunction^23^, but also cellular mechanisms are involved in developing arrhythmogenic pattern in the overt T2DM, such as early and delayed afterdepolarization, EAD and DAD, respectively^24^. Moreover, we also observed that apoptosis in FRD hearts was linked to mitochondria swelling and mitochondria membrane potential changes as well as to a closer distance between mitochondria and SR^22^.

Based on these results, we hypothesized that prediabetes-induced CaMKII SR-Ca^2+^ leak through RyR2 hyperactivity and increased SR-mitochondria tethering, alters mitochondrial metabolism and biodynamics, favoring fission processes. The present experiments were undertaken to explore this hypothesis, with the conviction that knowledge of the molecular aspects underlying the metabolic disturbances of cardiomyocytes in the prediabetic stage should be useful in developing strategies to prevent, avoid, or even reverse the structural and functional cardiac consequences of the overt disease.

## Results

### Increased cardiac RyR2 activity and SR-Ca^2+^ leak by FRD is prevented in CaMKII-genetically inhibited mice

**Figure 1:**
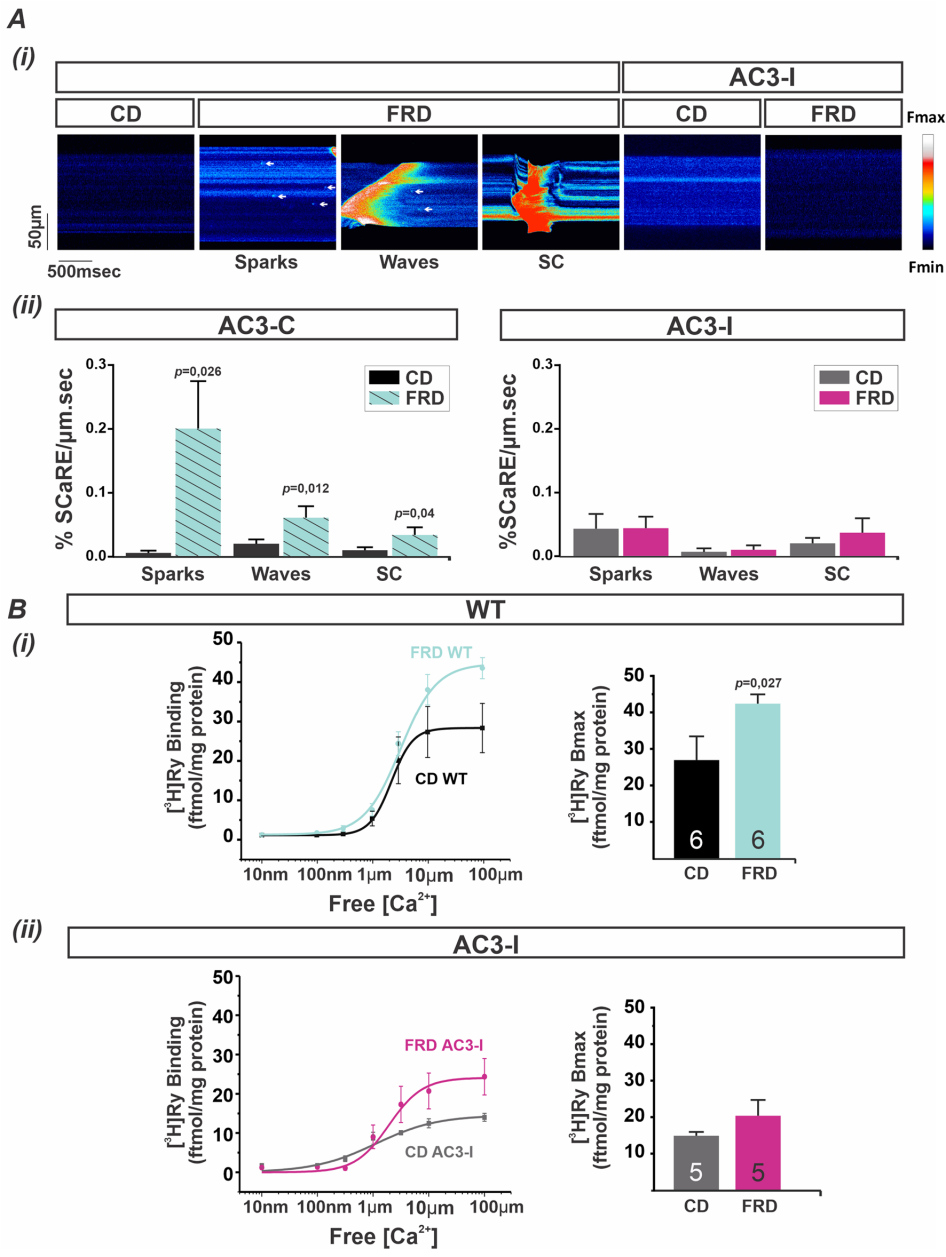
CaMKII-dependent RyR2 hyperactivity increases SR-Ca^2+^ leak in prediabetic mouse hearts. (**A**) (i) Representative confocal line scan images from CD and FRD AC3-C and AC3-I mouse cardiomyocytes showing sparks (white arrows), waves, and spontaneous contraction (SC), in FRD AC3-C mice. These spontaneous events are absent in CD WT and in CD and FRD AC3-I myocytes. (**ii**) Frequency of Spontaneous Ca^2+^ Release Events (SCaRE) in AC3-C and AC3-I mice. Mean ± SEM, n=10-13 cardiomyocytes from 3 mice per group. (**B**) Average [^3^H]Ryanodine binding curves fitted by Hill equation (left) and Bmax mean bar graph (right) from (**i**) CD and FRD WT mice and (**ii**) CD and FRD AC3-I mice heart homogenates. Only FRD WT mice present a significant increase in RyR2 maximal activity.

Recent findings of our group showed that phosphorylation of the S2814 site of RyR2 induces Ca^2+^ mishandling, increases apoptosis, and produces mitochondria-SR remodeling in mice fed with FRD (fructose-rich diet). This remodeling was suppressed in AC3-I mice, with cardiac-targeted CaMKII inhibition^22^. Since phosphorylation of the S2814 site increases RyR2 activity in a HEK293 cell line exposed to high glucose^22^, we hypothesized that AC3-I mice prevent the increased RyR2 activity and the induction of SR-Ca^2+^ leak. Confocal microscopy and [^3^H]Ry binding assay are shown in Fig. 1A and B suggest that this was the case, as was expected. Figure 1A (i) shows a typical recording of confocal images and (ii) average results revealing an increase in the frequency of SR-Ca^2+^ release events (SCaRE) in FRD AC3-C cardiomyocytes as compared to the CD AC3-C. In contrast, FRD treatment does not increase the SCaRE in cardiomyocytes from AC3-I mice (Fig. 1A (i) and (ii) right). Because AC3 (either C or I) mice co-express GFP as a reporter protein, it is crucial to measure intracellular Ca^2+^, due to GFP interference in Ca^2+^ signal, use the scramble mice (AC3-C) to compare with the CaMKII inhibited (AC3-I) mice. Moreover, the increased SR Ca^2+^ leak in FRD is the ignition step of the following events, even in rats^21^ and all the mice lines used previously^22^, we decide to continue the next experiments in WT mice (Please see discussion below). Figure 1B (i) shows a significant increment in [^3^H]Ry binding (B_max_) in FRD WT mouse hearts compared to CD WT hearts. Oppositely to WT littermate controls, AC3-I hearts exhibited a reduced B_max_ with CD and B_max_ did not significantly increase with FRD (Fig. 1B (ii)).

Taken together these data confirm the previous results^22^ and show that CaMKII inhibition prevents SR-Ca^2+^ leak and the increased activation of RyR2 produced by the prediabetic state. Given these findings, and results from our previous studies^22^, we next measured the prediabetic effects on mitochondrial parameters from FRD and CD WT mice heart, and then in FRD and CD AC3-I mice. These experiments were carried out to determine if these changes in RyR2 and SR-Ca^2+^ release CaMKII-dependent were associated with changes in the SR-mitochondrial ultrastructure, mitochondrial dynamics and metabolism.

### FRD increases SR-mitochondria proximity and expression of Mfn2, Grp75, and VDAC favoring mitochondrial Ca^2+^ load

**Figure 2:**
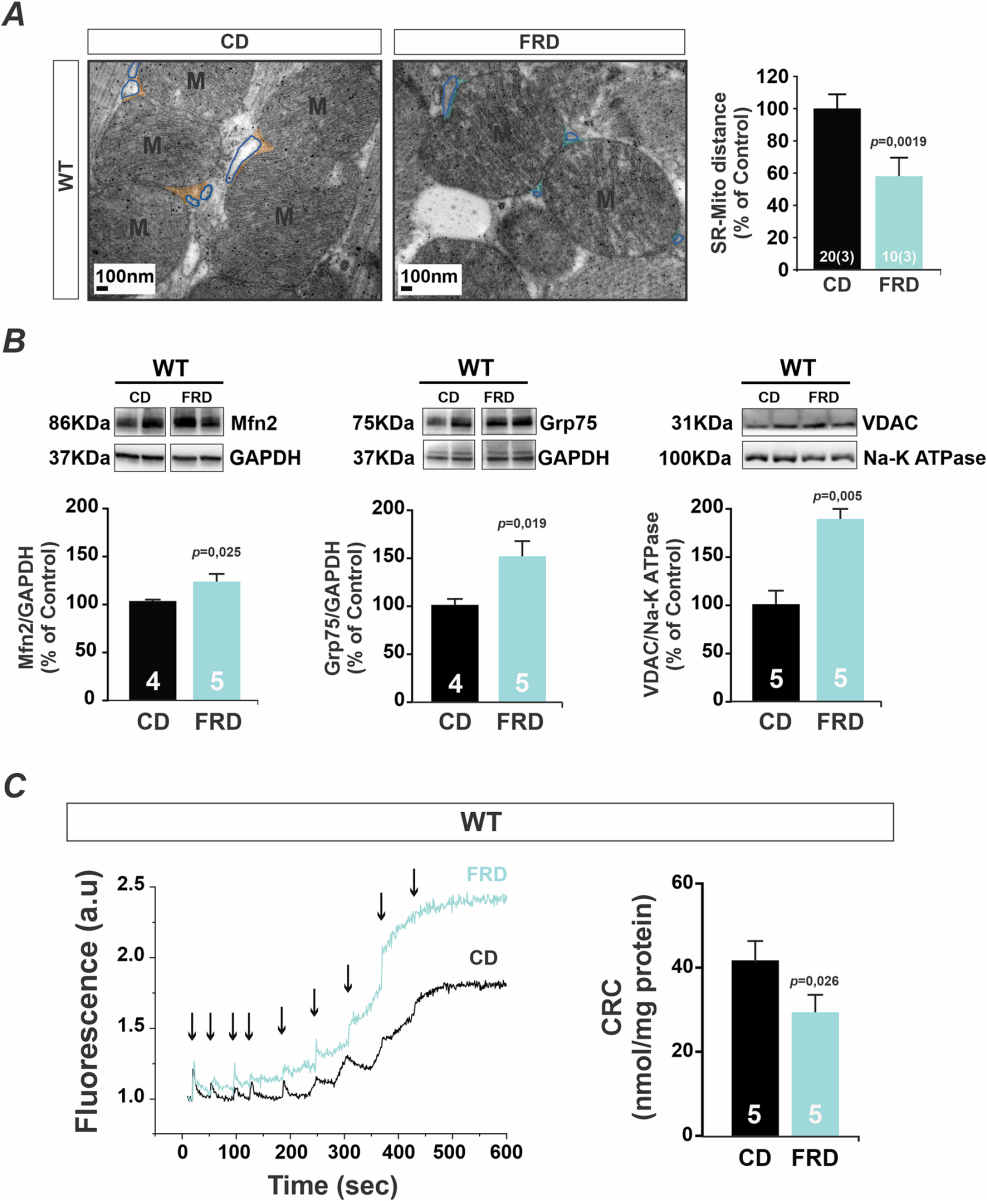
SR-mitochondria increased proximity and enhanced expression of tethering proteins favor mitochondrial Ca^2+^ retention capacity. (**A**) Left, representative skeletonized photographs of transmission electron microscopy (TEM) showing SR-mitochondria distance in hearts from CD and FRD WT mice. For the sake of clarity, the SR membrane was skeletonized with a blue line and SR-mitochondria distance was indicated in orange for CD and light blue for FRD WT specimens. On the right, average data of these results. There was a significant decrease in the distance between organelles in FRD with respect to CD WT mice. (**B**) Representatives immunoblots and average data of Mfn2, Grp75, and VDAC expression in CD and FRD WT mice heart. There is an increased expression of the three proteins examined in FRD vs. CD WT mice. (**C**) Ca^2+^ retention capacity (CRC) was measured by Calcium Green-5N salt fluorescence on isolated mitochondria suspension and normalized by protein content. Representative fluorescence traces (CD in black and FRD WT in light blue) are shown on the left. Arrows indicate 10µM Ca^2+^ pulses added to the suspension. On the right are shown the average data from these experiments. The CRC was calculated as the total Ca^2+^ uptake (number of peaks x 10µM) per mg of mitochondria protein. Mitochondria from FRD WT mice heart present a significant decrease in CRC with respect to CD WT mice.

It is well known that mitochondria and SR/ER (Sarcoplasmic/Endoplasmic Reticulum) are in tight contact^25^. In previous studies, we found that SR-mitochondria distance was decreased in prediabetic mouse cardiomyocytes by a mechanism at least partially mediated by CaMKII^22^. Confirming these previous findings, Fig. 2A shows a decrease in the distance between both organelles in FRD WT mice with respect to CD WT. Glucose-regulated protein 75 (Grp75)^26^ and Mfn2 are involved in determining the SR/ER-mitochondria contacts in mammals^12^. The junction SR/ER-mitochondria are faced where the mitochondrial membrane is abundant in voltage-dependent anion channel (VDAC), producing an ideal pathway for Ca^2+^ traffic between both organelles^14^. Therefore, we sought to investigate whether these proteins, Grp75, Mfn2, and VDAC, were involved in the prediabetic-induced increase in SR-mitochondria proximity.

Figure 2B shows that in FRD WT mice Mfn2, Grp75, and VDAC expression were increased compared to the CD WT. These results suggest that Mfn2, VDAC, and Grp75 could be involved in the increased SR-mitochondria proximity produced by FRD. Since increased SR-Ca^2+^ leak is associated with enhanced SR-mitochondria proximity, a condition anticipated to favor effective Ca^2+^ trafficking between both organelles, potentially could contribute to mitochondrial Ca^2+^ overload. To test this hypothesis, we performed experiments in isolated mitochondria to measure the Ca^2+^ retention capacity (CRC). Figure 2C shows representative recordings of CRC, where the decreased capability to take and keep extra-mitochondrial Ca^2+^ in isolated mitochondria from FRD mice compared to CD WT mice (left) was observed. These data could be the consequence of an increased MCU uptake and/or decreased NCLX (Na^+^/Ca^2+^/Li^+^ exchanger) Ca^2+^ efflux^27^. Overall results on the right show that mitochondria CRC was significantly higher in CD vs. FRD WT mitochondria. These results suggest that mitochondria Ca^2+^ load in FRD WT hearts is closer to the threshold at which mPTP could be opened, in agreement with the disturbance in mitochondrial membrane potential reported previously by our group^22^.

### Prediabetes triggers alterations in mitochondrial metabolism and morphology

Although optimal SR-mitochondria proximity is likely necessary and beneficial for mitochondrial function, it may turn to be detrimental, affecting mitochondrial properties as well as whole-cell metabolism under conditions of intracellular Ca^2+^ mishandling. Based on the differences in mitochondrial-SR ultrastructure and SR-Ca^2+^ handling, we next asked if FRD induced changes in mitochondrial metabolism, energetics, and morphology.

**Figure 3:**
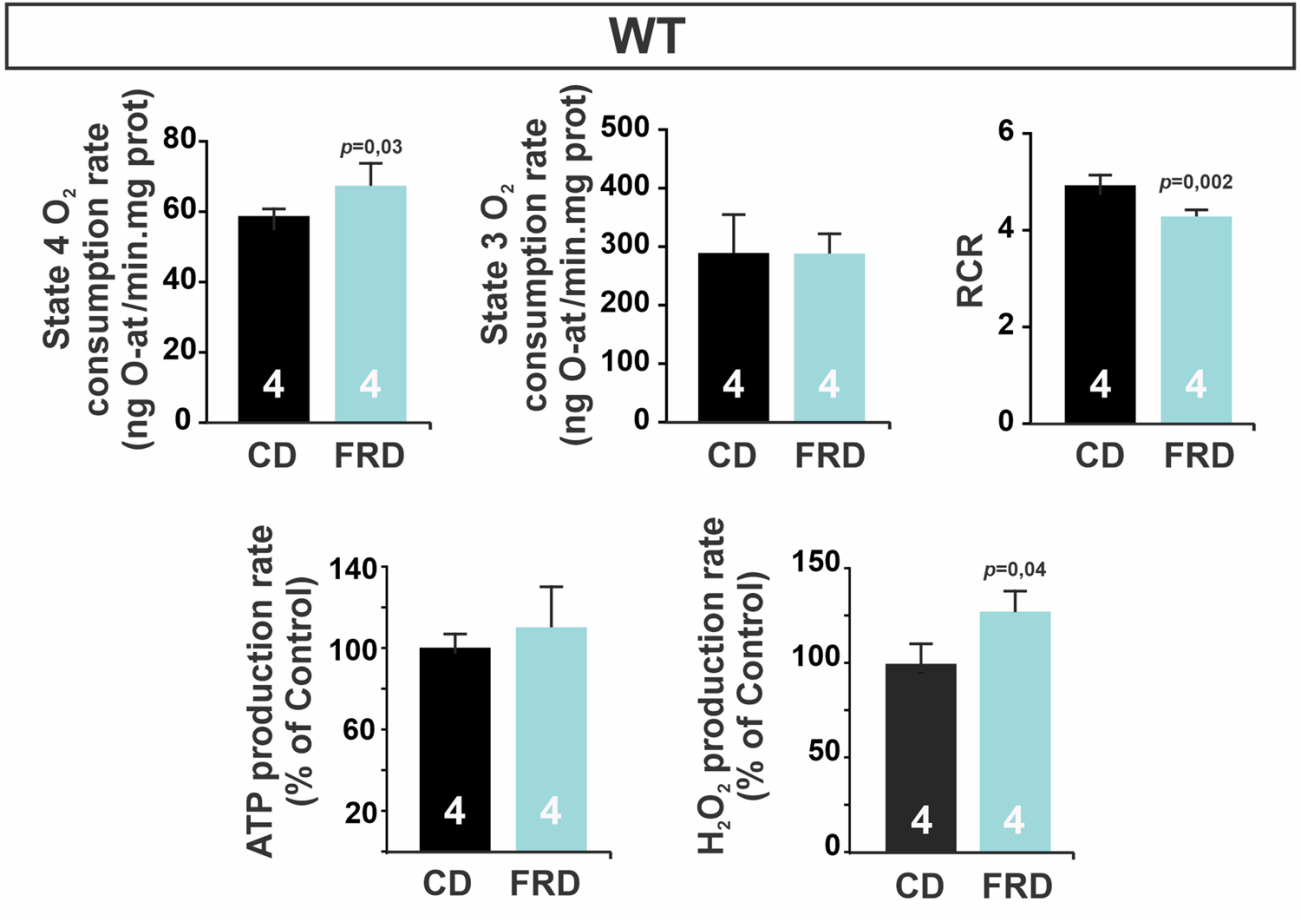
Mitochondrial metabolism is altered in prediabetic mice. The upper panel shows mitochondrial O_2_ consumption rate in isolated mitochondria in State 4 (without extra ADP and excess of substrates), State 3 (with the addition of ADP), and Respiratory Control Ratio (RCR). RCR was calculated as a State 3/State 4 respiration rate. The bottom panel shows ATP and H_2_O_2_ production rates expressed as % of CD group. All measurements were performed on isolated mitochondria using malate and glutamate as substrates. O_2_ consumption in State 4 and H_2_O_2_ production were increased, while RCR is decreased in FRD vs CD WT mitochondria, indicating a mild ETC uncoupling.

We measured mitochondrial O_2_ consumption, ATP, and H_2_O_2_ production rate in freshly isolated mitochondria. In WT mice, FRD significantly increased mitochondrial O_2_ consumption in State 4 (resting respiratory state, without ADP addition) without significant changes in State 3 (active respiratory state, with ADP addition) when compared to CD (Fig. 3). Supporting these findings respiratory control rate (RCR), the ratio between State 3 and State 4, representing mitochondrial integrity and coupling, was decreased in FRD as compared to CD (RCR in CD = 4.9 ± 0.2 vs. FRD = 4.3 ± 0.2; *P* < 0.01) (Fig. 3, upper panel). Mitochondrial ATP production rate showed no differences between FRD and CD (Fig. 3, bottom panel). Moreover, FRD WT mice showed an increase in mitochondrial H_2_O_2_ production rate as compared to CD (Fig. 3, bottom panel). Taken together, the associated increase in O_2_ consumption in State 4, the decreased RCR and the increased mitochondrial H_2_O_2_ production rate would suggest that a percentage of O_2_ is reduced to form superoxide anion (O_2_^·−^), which rapidly dismutates to H_2_O_2_^28^. These findings reveal a mild uncoupling of electron transport change (ETC), likely responsible for the increase in H_2_O_2_ production through O_2_^·−^ dismutation^29^.

**Figure 4:**
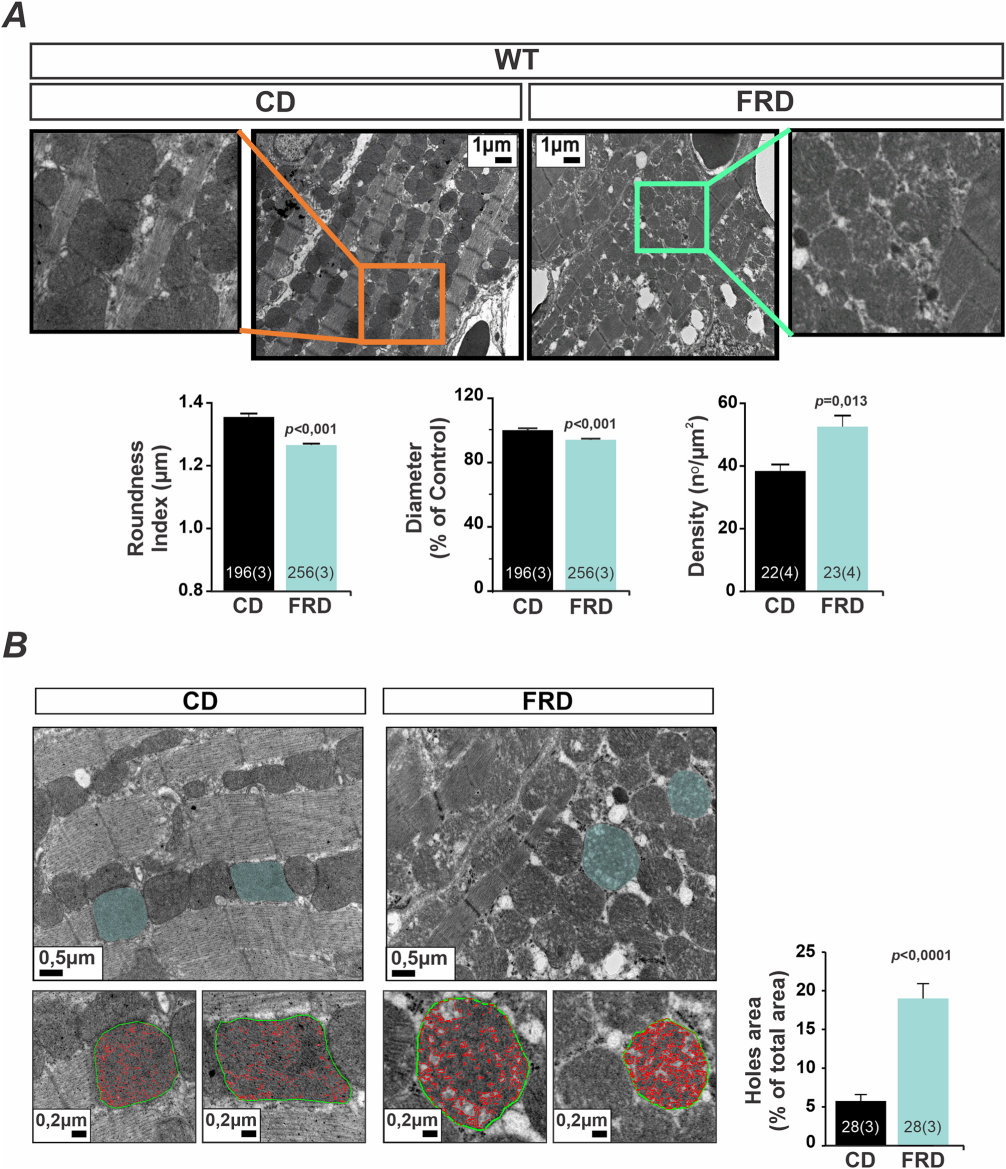
Mitochondria morphology and density are altered in prediabetic mouse hearts. (**A**) Representative transmission electron microscopy (TEM) photographs from cardiac tissue and average bar graphs of mitochondria morphology parameters (Roundness and Diameter) and mitochondria density. FRD WT presents a decrease in diameter and an increased roundness and density with respect to CD WT specimens. (**B**) Representative TEM photographs of hearts from CD and FRD treated mice, showing the analysis developed to measure the area of holes in the mitochondria selected (light blue area). The magnified photographs below show the skeletonized mitochondria (green line) and the holes area (red line). The bar graph shows the average of holes area normalized by the entire mitochondria area. The mitochondria from FRD WT present increased holes area with respect to CD WT mice heart.

To investigate whether the altered mitochondria metabolism of prediabetic hearts was associated with changes in mitochondrial morphology, we use transmission electron microscopy (TEM) from CD and FRD WT hearts specimens. Figure 4A shows that FRD generated remarkable morphological alterations in mitochondria of WT mice. We found a significant decrease in mitochondria size and mitochondria roundness index (where 1 is equal to a sphere), and an increase in mitochondria density in FRD vs CD WT mice. Interestingly, the heart tissue from FRD WT mice exhibited disarray in mitochondria and myofibril distribution that, although difficult to quantify, were consistent in all microphotographs explored (Supplementary Figure S1-A). This tissue disorder was associated with a higher number of vacuoles^30^ (Supplementary Figure S1-A), which could be the result of the increased mitochondria fission process (at the final state)^30^. Besides, mitochondria from FRD hearts showed visible disruption in the structure, evidenced as holes or less electrodense areas, that were significantly higher than CD mitochondria (Fig. 4B). The findings suggest that these alterations are in agreement with the previous results where we detected mitochondrial swelling by optical density in FRD WT isolated mitochondria^22^.

**Figure 5:**
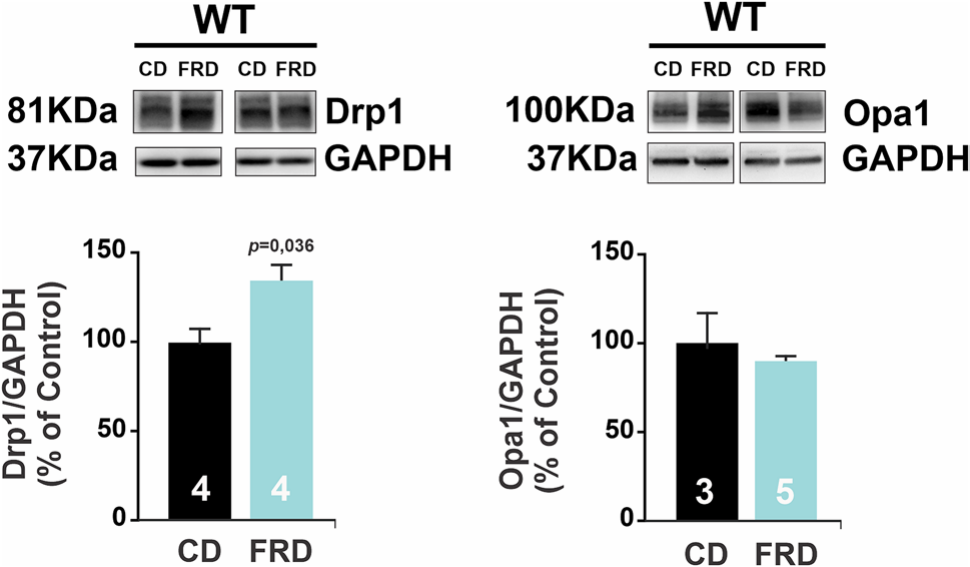
Prediabetes increases in fission protein expression in the heart. Representative immunoblots of Drp1 and Opa1, fission and fusion proteins, respectively, and average bar graphs. FRD WT mice heart has an increase in Drp1 but not in Opa1 expression with respect to CD WT.

The decreased mitochondrial size, the increased mitochondrial density, and the impairment of mitochondrial metabolism described above strongly suggested the occurrence of mitochondrial fragmentation, which in turn induces vacuole formation. The fragmentation may result from increased fission, decreased fusion, or both. Opa1 and Mfn1/2 have been described to be essential for mitochondrial fusion, whereas Drp1 is considered the “master regulator” of mitochondrial fission, responsible for constricting the mitochondrial membrane^10,16^. We, therefore, investigated whether these proteins were modified in FRD WT hearts. Figure 5 shows that there was an increase in Drp1 expression in FRD WT compared to CD WT mice. In contrast, Opa1 expression was unaltered. Although, as previously shown in Fig. 2, Mfn2 expression was increased in FRD WT vs. CD hearts, the association between mitochondria fragmentation, increased expression of Drp1 and the altered mitochondrial structure in FRD vs CD, indicate an exacerbated fission over fusion process in these hearts. In this context overexpression of Mfn2, although is associated with the fusion process^10,11^, would be involved in its non-canonical function as a tethering protein between SR and mitochondria^12–14^.

### Genetic inhibition of CaMKII prevent changes in SR-mitochondria distance and CRC in prediabetic mice heart

**Figure 6:**
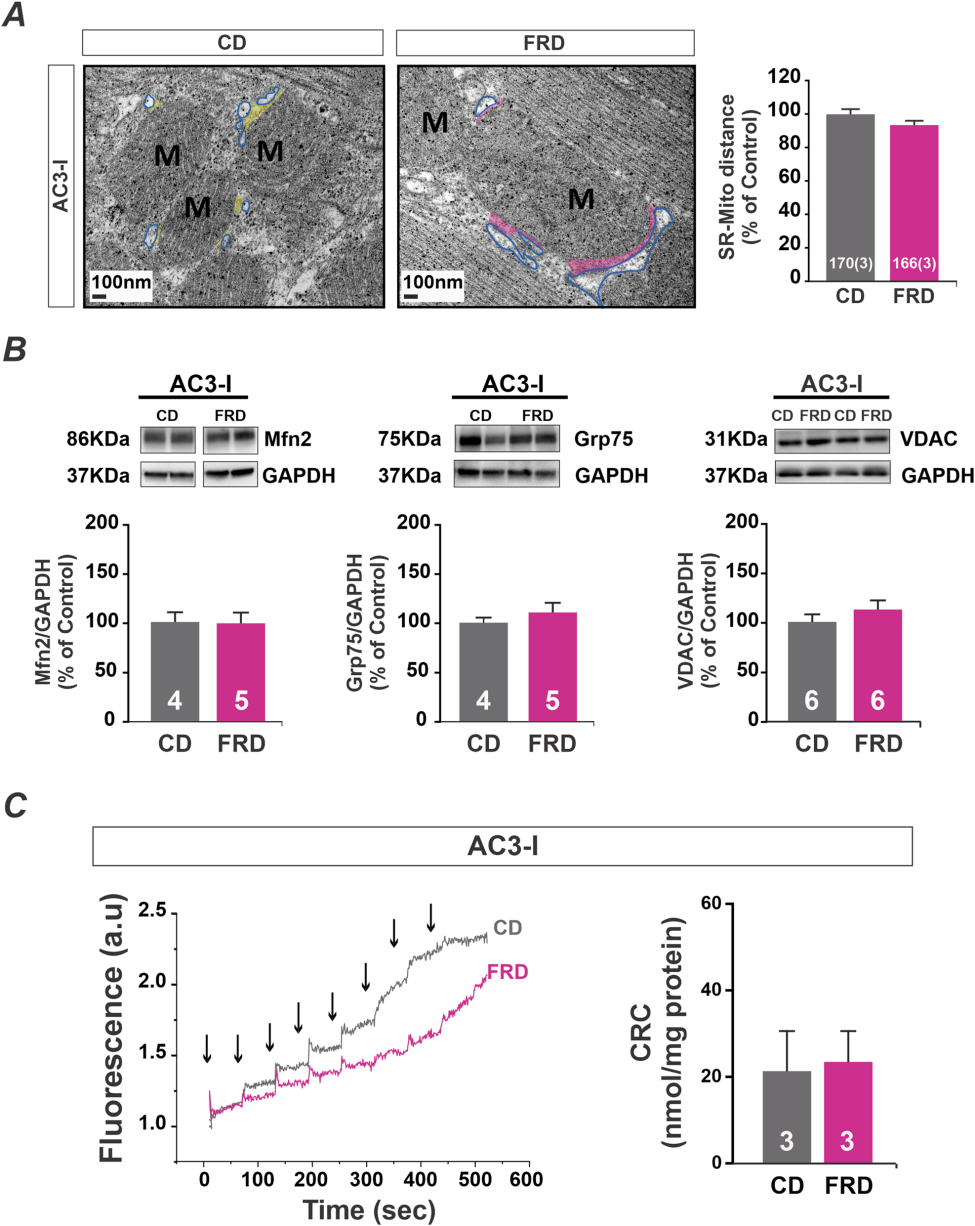
Genetic CaMKII inhibition prevents SR-mitochondria increased proximity avoiding mitochondrial Ca^2+^ retention capacity in prediabetic mice heart. (**A**) Left, representative skeletonized photographs of transmission electron microscopy (TEM) showing that AC3-I mice heart samples are protected from decreased SR-mitochondria distance induced by FRD. The SR membrane was skeletonized with a blue line and SR-mitochondria distance was indicated in yellow for CD and pink for FRD AC3-I specimens. On the right, average data of these results. (**B**) Representatives immunoblots and average data of Mfn2, Grp75, and VDAC expression in CD and FRD AC3-I mice heart. Similar results were obtained in FRD and CD AC3-I mice. (**C**) Ca^2+^ retention capacity (CRC) representative fluorescence traces from AC3-I mitochondria (CD in gray and FRD in pink) are shown on the left. Arrows indicate Ca^2+^ pulses addition to the suspension. On the right, the average data from these experiments shows no differences between CD and FRD AC3-I mitochondria.

The distance between SR and mitochondria was not significantly changed by FRD in AC3-I mice (which express the CaMKII inhibitor peptide AC3 at heart level) with respect to the CD (Fig. 6A). Accordingly, the expression of the protein Grp75, VDAC, and Mfn2 was similar in FRD and CD AC3-I mice (Fig. 6B). These results indicate that the decreased distance and increased expression of proteins as Mfn2, VDAC, and Grp75 could be the consequence of CaMKII activation. Since the proximity of SR to the mitochondria in WT mice affected the CRC, we measure this parameter in isolated mitochondria from FRD and CD AC3-I mice. The CRC did not decrease in FRD AC3-I with respect to the CD mitochondria, indicating prevention of mitochondria Ca^2+^ overload (Fig. 6C). Taking together, these results suggest that the prevention of altered SR-mitochondria communication, observed in FRD WT mice, is avoided by CaMKII inhibition.

### Inhibition of CaMKII prevents altered mitochondria metabolism and morphology induced by FRD

**Figure 7:**
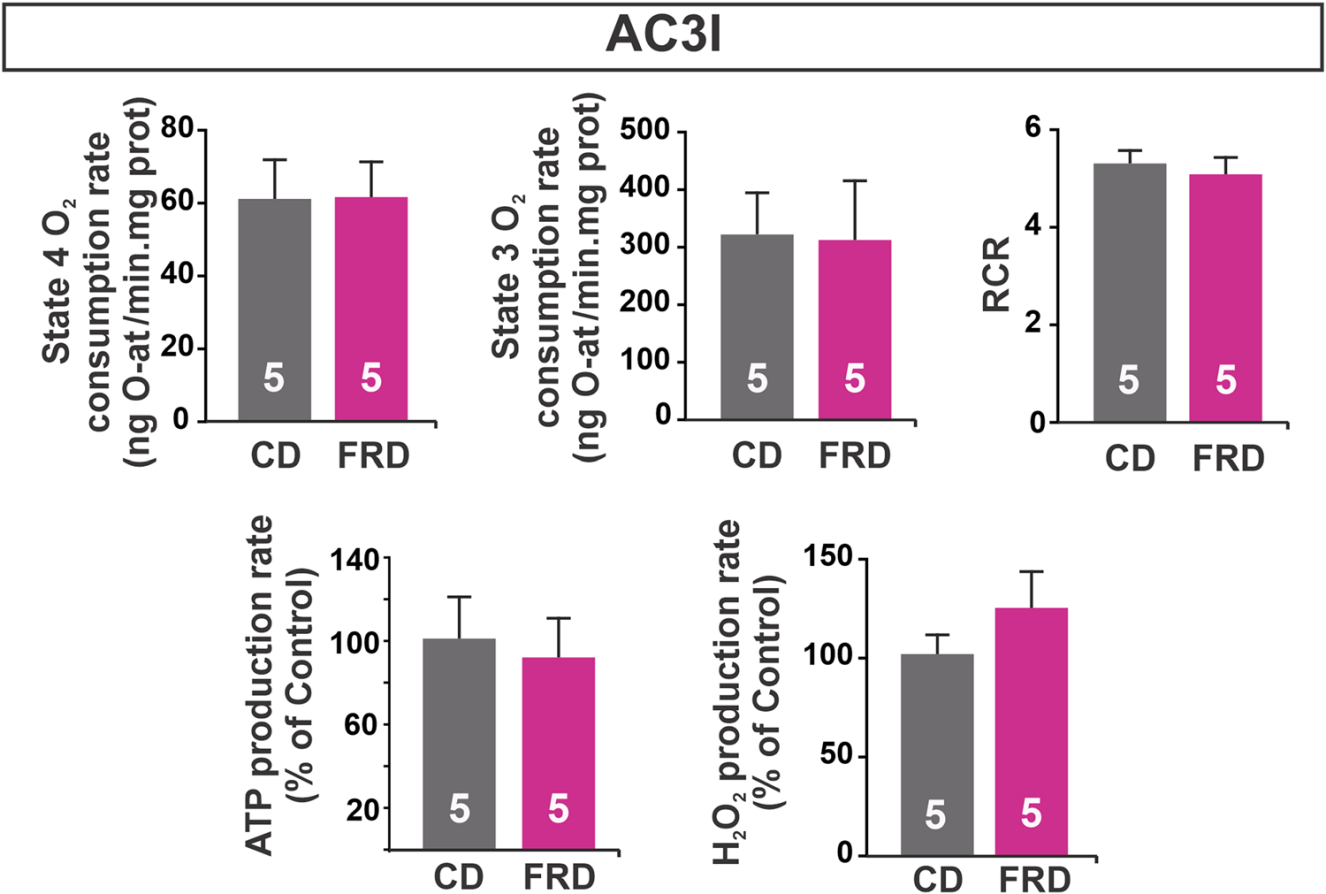
Mitochondrial metabolism is preserved in CaMKII inhibited prediabetic mice. The upper panel shows mitochondrial O_2_ consumption rate in isolated mitochondria in State 4 (without extra ADP and excess of substrates), State 3 (with the addition of ADP), and Respiratory Control Ratio (RCR). RCR was calculated as a State 3/State 4 respiration rate. The bottom panel shows ATP and H_2_O_2_ production rates expressed as % of CD group. All measurements were performed on isolated mitochondria using malate and glutamate as substrates. All the parameters measured were similar in FRD and CD AC3-I mitochondria.

In FRD WT mice the mitochondria presented a mild un-coupling of ETC related to increased H_2_O_2_ with no changes in ATP production rate. In contrast, mitochondria bioenergetics were not altered by FRD when compared to the CD group in AC3-I mice (Fig. 7). These results suggest a CaMKII-mediated alteration in mitochondrial metabolism in prediabetic animals.

**Figure 8:**
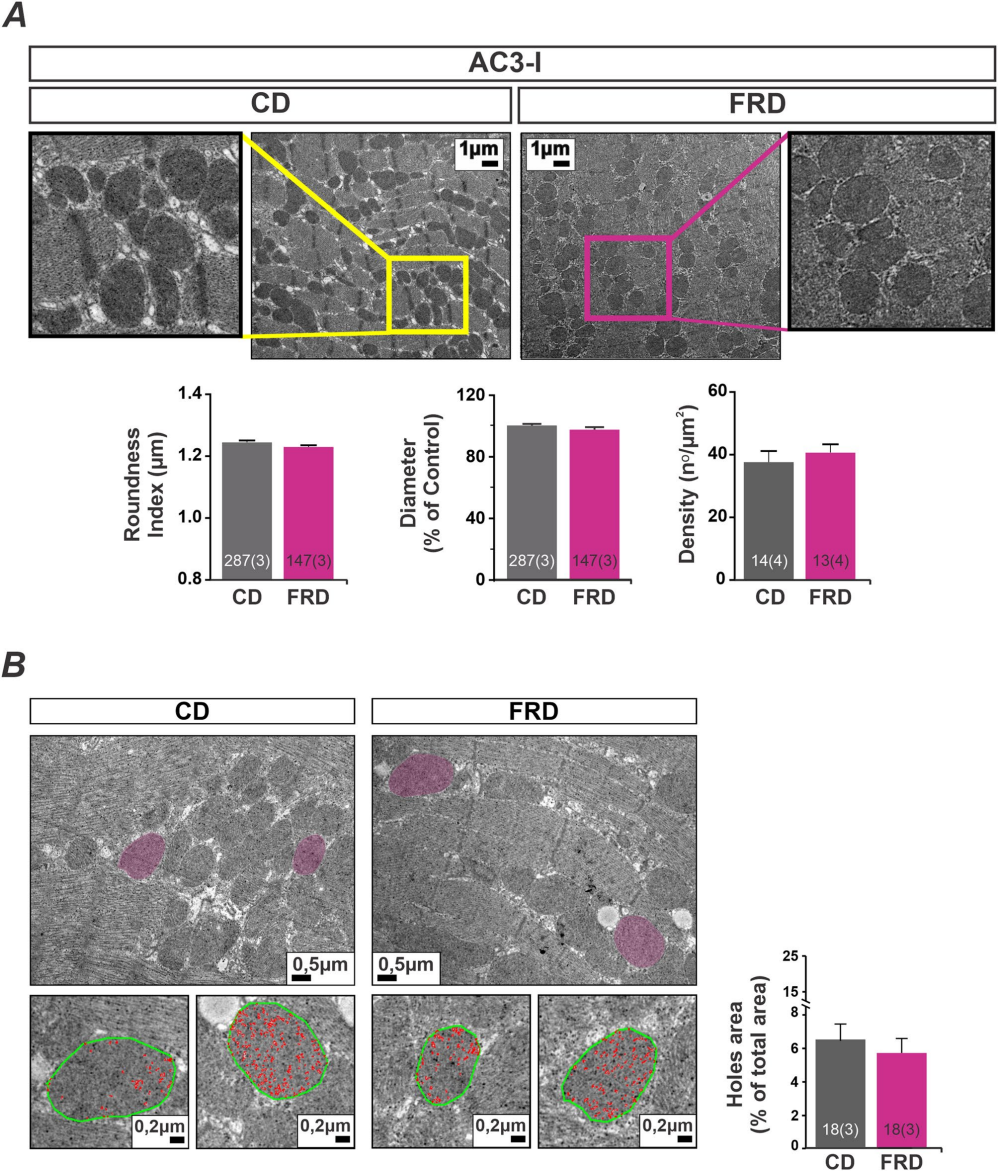
Altered mitochondria morphology and density in prediabetic mouse hearts are prevented with genetic CaMKII inhibition. (**A**) Representative transmission electron microscopy (TEM) photographs from cardiac tissue, and average bar graphs of mitochondria morphology parameters (Roundness and Diameter) and mitochondria density. FRD did not modify the mitochondria morphology in AC3-I mice samples. (**B**) Representative TEM photographs of hearts from CD and FRD treated AC3-I mice, showing the analysis developed to measure the area of holes in the mitochondria selected (pink area). The magnified photographs below show the skeletonized mitochondria (green line) and the holes area (red line). The bar graph shows the average data indicating that mitochondria structure from AC3-I mice heart is similar in CD and FRD.

**Figure 9:**
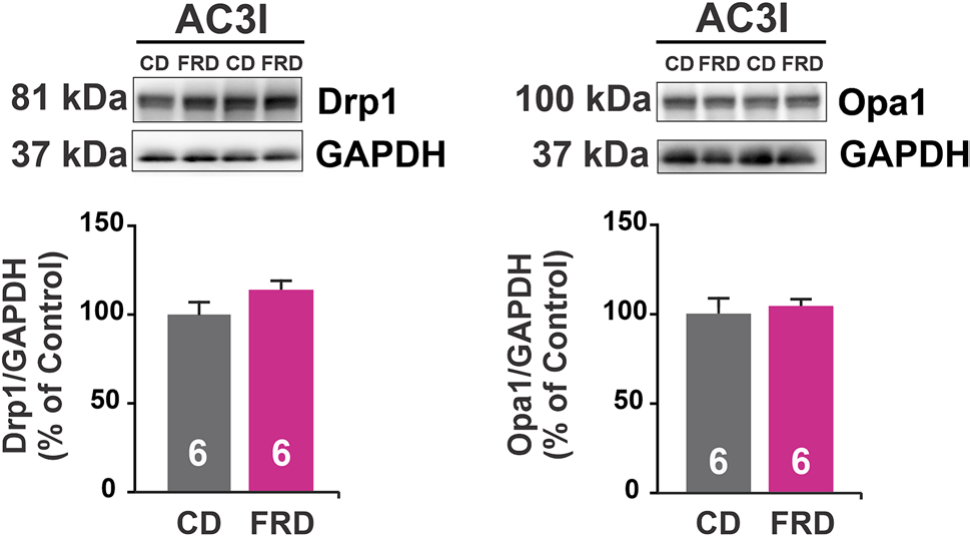
Cardiac CaMKII inhibition precludes unbalanced dynamics induced by prediabetes. Representative immunoblots of Drp1 and Opa1, fission and fusion proteins, respectively, and average bar graphs. FRD and CD AC3-I mice heart present similar levels of Drp1 and Opa1 expression, suggesting a balanced mitochondria fusion and fission processes.

The mitochondria metabolism and energy are in close relation with mitochondria morphology, therefore we wanted to confirm whether CaMKII inhibition is involved in FRD induced mitochondria shaping. We measure mitochondria morphology by TEM photographs, and there was no difference in diameter, roundness, and density induced by FRD in AC3-I mice (Fig. 8A). Also, the presence of holes inside the mitochondria was similar between FRD and CD AC3-I mice (Fig. 8B). Besides, fission/fusion proteins expression was comparable in FRD with respect to CD AC3-I mice (Fig. 9) suggesting that CaMKII is involved in this remodeling process.

The absence of all these events in FRD AC3-I mice suggests the pivotal and deleterious role of the CaMKII pathway activation in prediabetic heart alterations.

## Discussion

Ca^2+^ mishandling is responsible for several diseases that take place at the level of different tissues along our body, including tumorigenesis (cell proliferation and migration), autoimmune, and neurodegenerative diseases. In the heart, Ca^2+^ is particularly indispensable for muscle contraction but also for energy production as a cofactor of NAD dehydrogenases. Therefore, Ca^2+^ handling is finely tuned and its alterations, even at restricted subcellular microdomains, may trigger cardiac remodeling which can evolve to heart failure (HF) and arrhythmias. Indeed, intracellular Ca^2+^ depletion leads to impaired contractility while an excess of Ca^2+^ activates phosphatases and kinases involved in reprogramming gene transcription and unbalancing reactive oxygen/nitrogen species production which may give rise to malignant arrhythmias and cell death.

We previously described that prediabetic hearts present arrhythmias and apoptosis associated with Ca^2+^ mishandling^21,22^. In the present study, we moved forward to further investigate the mechanisms by which Ca^2+^ mishandling defects alter cardiac function in prediabetic hearts. We hypothesized that the FRD-induced CaMKII-dependent increase in SR-Ca^2+^ leak promotes a dysregulated interplay between Ca^2+^ handling and mitochondrial bioenergetics that may constitute the first steps to cardiac failure in the diabetic heart. Our results revealed that SR-Ca^2+^ leak decreases mitochondria Ca^2+^ buffering, through a CaMKII dependent activation of RyR2. It was further shown that there is a CaMKII dependent ultrastructure remodeling at the mitochondria-SR microdomain which reduces the distance between these organelles and may contribute to increasing mitochondria Ca^2+^ load. This study also provides evidence that CaMKII activity and the enhanced mitochondria Ca^2+^ load are involved in mitochondria metabolism alterations and the increased fission process present in prediabetic hearts.

### The Ca^2+^ highway from SR to the mitochondrion in prediabetes

The present results confirm that FRD evokes an enhanced SR-Ca^2+^ release in association with increased proximity of SR-mitochondria and that both alterations are mediated by CaMKII. Yet, the molecular basis of this increased interaction is not known. Ca^2+^ transfer from SR/ER to mitochondria is a well-characterized function of tethering zones. Localized Ca^2+^ released from the SR/ER stimulates mitochondrial Ca^2+^ uptake. VDAC at the OMM, and principally MCU at the IMM, drive Ca^2+^ from microdomains to the mitochondrial matrix^27,31,32^. Moreover, in different tissues, Grp75 is required to form VDAC/Grp75/IP3R channel complexes^33,34^. Mfn2, which resides at the OMM, is essential (canonically) for mitochondrial fusion^10^, but also has been reported to be expressed in the ER, forming ER-mitochondrial tethers via homodimerization or heterodimerization with Mfn1 at the OMM (non-canonical role)^12^. In addition, other studies have elegantly shown that in the absence of Mfn2, the enhanced distance ER-mitochondria, slowed mitochondria Ca^2+^ uptake^12,35^. Supporting the essential role of this inter-organelle communication, Seidlmayer et al.^36^ showed that when the physical link between SR and mitochondria by Mfn2 was disrupted in ventricular cardiac myocytes of Mfn2 KO mice, the SR-mitochondrial metabolic feedback mechanism and ATP production were highly compromised. Similar results were obtained in Grp75 KO mice^37^. In line with these previous findings, the present study demonstrated that Mfn2, VDAC, and Grp75, all proteins critically involved in SR/ER-mitochondria interaction, are overexpressed in prediabetic WT mice (FRD treated mice) by a mechanism also dependent on CaMKII. Our results, therefore, indicate that the enhanced SR-mitochondria proximity observed in FRD WT hearts, further increases Ca^2+^ trafficking (already favored by the enhanced CaMKII-dependent SR-Ca^2+^ leak), between both organelles. Since the mitochondrial Ca^2+^ uptake CaMKII-dependent through the MCU is controversial^38–41^, we cannot ensure that CaMKII is responsible for MCU activity. In fact, Luczak et al.^39^, have recently described that the AC3-I mice do not express the inhibitory peptide inside the mitochondria. If mitochondrial CaMKII (mtCaMKII) was activating the MCU complex, FRD AC3-I mice should have mitochondrial Ca^2+^ overload with respect to CD AC3-I. In this context, increased Ca^2+^ at the microdomains by SR-Ca^2+^ leak is responsible for decreased mitochondrial CRC. Therefore, we propose that the increased Ca^2+^ levels at the SR-mitochondria microdomains, in addition to the increased proximity between organelles, favors the mitochondrial Ca^2+^ uptake and consequently, disturbing mitochondrial function. However, further investigations are needed to identify the role of mitochondrial transporters, as MCU complex, mitochondrial RyR1, and NCLX^42–45^, that could participate in mitochondrial Ca^2+^ overload in prediabetic model. Despite there have been described that the MCU is downregulated in DM^46,47^, in others pathologies, as heart failure, its inhibition provide cardioprotection^48^. Nevertheless, mitochondrial Ca^2+^ handling in prediabetic models is an unexplored field.

### The pathway from mitochondrial Ca^2+^ overload to the fission process: oxidative stress role

The present results reveal that prediabetes increases mitochondria fission. Indeed, the significant decrease in mitochondria size and increase in mitochondria sphericity and density observed in FRD WT mice associated with the Drp1 overexpression, greatly support this contention. Significantly, both phenomena, mitochondrial morphological changes and Drp1 overexpression did not occur in FRD AC3-I mice, in which CaMKII activity was absent. These data support the notion that CaMKII is involving in Drp1 overexpression and mitochondria fission in prediabetic hearts. The results are consistent with previous *in vitro* evidence suggesting that hyperglycemia induces mitochondrial fragmentation and mitochondrial ROS accumulation in H9c2 cells, a process that was suggested to be Drp1-dependent and that would lead to cell death by apoptosis^49^. Besides, mitochondrial fragmentation was associated with tissue disorder and a higher number of vacuoles in FRD vs CD WT mice, which is not evident in FRD vs CD AC3-I mice. The vacuole formation has been linked to a final state of mitochondria degradation, which begins with an IMM compromised (cristae onion-like), continues with OMM alterations and finally, the entire degeneration of IMM and OMM results in vacuole formation^30^.

Mitochondria fission is related to an impaired ETC and F_1_-F_0_-ATPase activity. In this scenario, it has been shown that mitochondria dynamics are relevant to different physiological as well as pathological processes, like HF, T2DM, and apoptosis. In previous experiments, we demonstrated that an increase in apoptosis was also a prominent finding in prediabetic hearts^22^. Indeed, mitochondria are the primary source of ATP but also of ROS production, which in turn can trigger oxidative stress, thereby determining the fate of the cell (survival or death)^50^. The rate of ETC is Ca^2+^-dependent, which activates the TCA cycle^51^, generating NADH and activating ATP^52^, and ROS production that are eliminated by different mechanisms^7^. This mitochondrial triangle Ca^2+^-ATP-ROS is highly regulated and any misbalance in one of them may result in changes in the other two^53^. The increased O_2_ consumption and decreased RCR in prediabetic mice indicate that ETC is highly demanded with the acceleration of NADH and NADPH consumption. This process may have two possible consequences: (1) Increased O_2_ consumption and H^+^ extrusion with a rise in the rate of ATP production if the ETC is coupled or (2) Increased O_2_ consumption and H^+^ extrusion without any associated ATP production and enhanced O_2_^**·**−^ and H_2_O_2_ generation if the ETC is un-coupled^7,54^. Our results strongly suggest that the second one is the case of prediabetic hearts; the increase in SR-Ca^2+^ leak and the enhanced SR-mitochondria proximity, are responsible for the decreased mitochondrial CRC observed, which in turn may trigger the increased H_2_O_2_ production described, unbalancing ROS production and elimination, typical of oxidative stress. Accordingly, in previous results, we have demonstrated that an increase in lipid peroxidation was associated with apoptosis and arrhythmias^21,22^. Moreover, scavenging ROS avoids CaMKII activation and, therefore, apoptosis^22^. It worth to mention that either increased Ca^2+^ or ROS can generate mPTP opening, and that have been described in several pathological conditions^36,55^. In fact, the decreased CRC and increased holes area in mitochondria are related to previous results in which prediabetic mitochondria swells^22^. Altogether, these results reveal the major role of mitochondrial-ROS production on prediabetic heart pathogenesis, non-previously demonstrated.

### Prediabetes and alarm signs: from molecular aspects to clinical disease

Prediabetes has a growing prevalence and worldwide incidence^56,57^. However, although cardiac pathophysiological alterations are relatively well characterized in fully developed diabetes (i.e., diabetic cardiomyopathy), information about prediabetes is limited due to its poor diagnosis. Cardiac dysfunction in diabetic cardiomyopathy has been attributed to numerous causes and different pathways (i.e., increased oxidative stress or activated apoptosis^58,59^, impaired mitochondrial function^60^, autophagy^59^, and imbalance of Ca^2+^ homeostasis^61^). Although it has been described that the prediabetic state induces mild diastolic dysfunction, cardiac consequences of prediabetes and their molecular mechanism are still unclear. The prediabetic model used in the present study was validated by previous works from our^21,22^ and other laboratories^19,20^, and the period of treatment to achieve this model is extremely short (21 days). Therefore, the findings reported in this and the previous studies^21,22^ seem to occur very early in the disease’s development. These facts imply that the pathways running in the overt diabetic cardiomyopathy started when glucose mishandling was just at the beginning, emphasizing the emergency of routinely testing of prediabetes in the population. Actually, health organizations have not uniform criteria for screening prediabetes in individuals that do not present risk factors for T2DM (obesity, hypertension, familiar background, etc)^62^; although this is not a translational study, our prediabetic mice model did not show any apparent comorbidity to suspect prediabetes but did show several and serious heart perturbations. The results from this and our previous studies indicate that prediabetes is a silent process, with activation of harmful molecular pathways, extremely aggressive from the beginning, that should be stopped on time to prevent the progression to a more deleterious stage of the disease.

### CaMKII as a central player in prediabetes

As we mentioned before, several cardiac alterations in diabetic cardiomyopathy have been attributed to CaMKII activation^24,63,64^. In this regard, Luo *et al.*^63^ described that oxidized CaMKII (ox-CaMKII) was significantly increased in pacemaker tissues from diabetic patients compared with that in non-diabetic patients after myocardial infarction. In that work, the authors described that Streptozotocin-treated mice had increased pacemaker cell ox-CaMKII and apoptosis, which were further enhanced by myocardial infarction^63^. Several years before, in 2007, the group of Xiao^65^ already described that CaMKII is a central key of several apoptotic stimuli that activates cell death pathways in cardiac muscle. In addition, CaMKII activation by oxidation seems to be a redox sensor that determines health and on-off disease switch, as well the sensitivity to ROS levels across the life span^66^. Moreover, other authors attribute CaMKII-deleterious effects over myocardium through phosphorylation of histone deacetylases^67,68^ and to the activation of other kinases (PKD, PKA)^67,69^. PKD is a member of the CaMK superfamily and has been reported to be inhibited by AC3-I^67^; therefore we could not discard a PKD effect in our results. Nevertheless, in previous results from our group^21,22^ we found several harmful effects in the heart due to ox-CaMKII hyperactivity. Those effects were prevented in SR-AIP and S2814A, two mice lines that specifically presents the lack of CaMKII phosphorylation of its targets at SR downstream the kinase activation. The previous results are beyond PKD inhibition and support the data present in the actual work in AC3-I mice. The role of CaMKII in prediabetes has taken a relevant place just in the last years. In our laboratory, using this short-term treatment of fructose-rich diet (FRD), we discovered that prediabetic mice present ventricular arrhythmias that were dependent on CaMKII phosphorylation of RyR2^21^. In the scenario of FRD treatment, CaMKII is activated by increased oxidative stress ^21^, although we do not discard other ways of activation that could be masking the oxidation of the enzyme, i.e. *O*-GlcNAcylation^64^. Later, we described that the same pathway produces apoptosis in the prediabetic heart involving the participation of mitochondria^22^. In the present study, we continue characterizing the prediabetic heart and revealed that CaMKII is also involved in connecting the SR-Ca^2+^ leak to the mitochondria Ca^2+^ overload and disturbed mitochondria metabolism. Moreover, mitochondria fission observed in prediabetic hearts is also linked with CaMKII activation. Once more, CaMKII hyperactivity results in deleterious outcomes to the heart function apparently not compensated by other mechanisms that may counteract these hazardous effects.

## Conclusions and limitations

**Figure 10:**
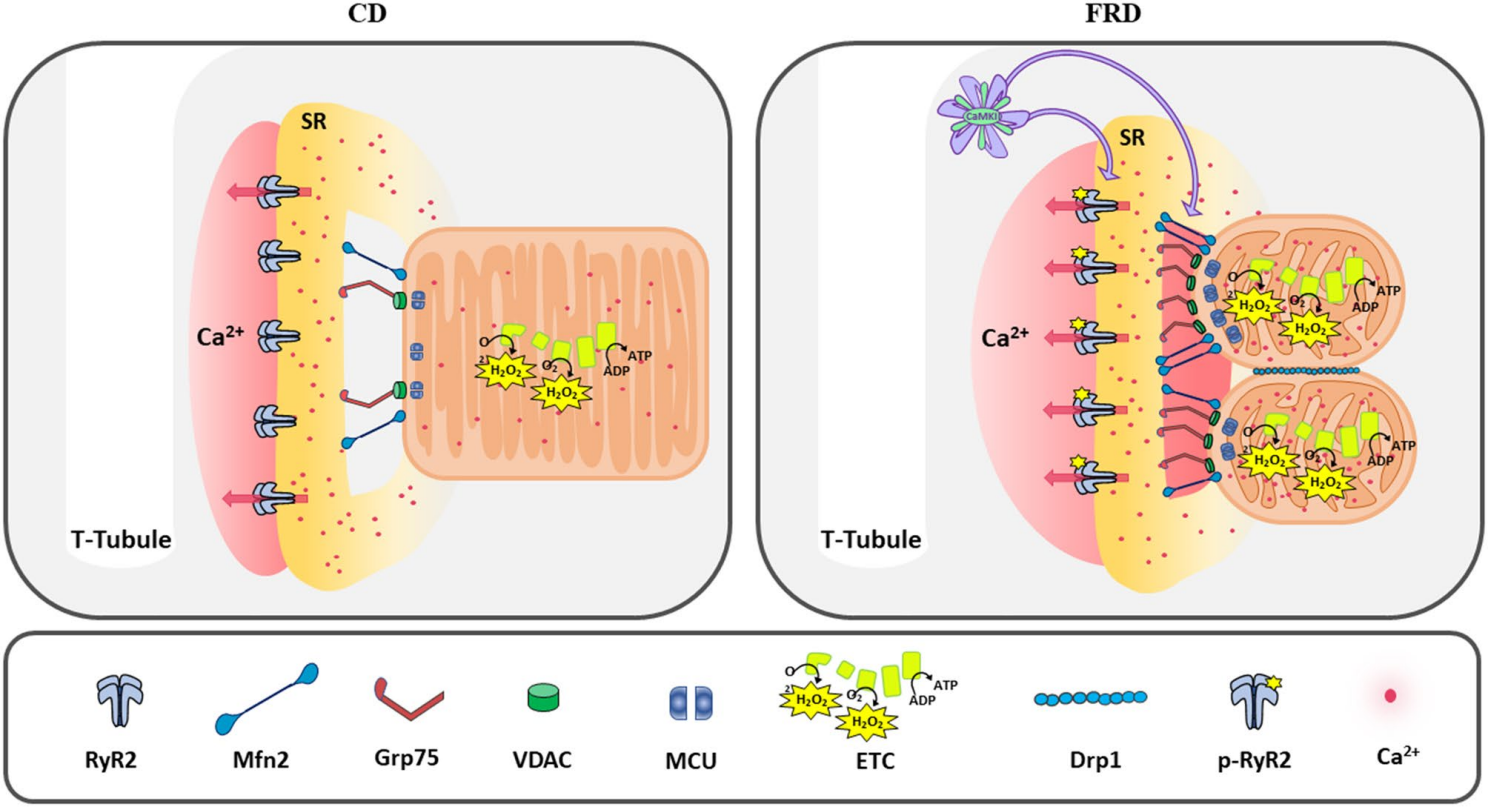
Conclusion and discussion cartoon. Scheme representing the CaMKII pathway of mitochondria alterations produced by prediabetes (right) with respect to normal CD (left). CaMKII, Ca^2+^-Calmodulin kinase II. SR, sarcoplasmic reticulum. RyR2, ryanodine receptor 2. Grp75, glucose-regulated protein 75. Mfn2, Mitofusin 2. VDAC, voltage-dependent anion channel, MCU, mitochondrial Ca^2+^ uniporter. Drp1, dynamin-regulated protein 1. ETC, electron transport chain.

In the present work, we have described for the first time that as earlier as in a prediabetic state, the heart SR-mitochondria microdomains suffer a CaMKII remodeling, involving SR-Ca^2+^ leak and mitochondria fission. In Fig. 10 we schematize the events in prediabetic mice hearts. CaMKII hyperactivity induces SR-Ca^2+^ leak by RyR2 activation in FRD AC3-C mice, which in turn decreases mitochondrial CRC due to, at least in part, the enhanced SR-mitochondria tethering. Although these experiments indicate an increased mitochondrial Ca^2+^, further precise experiments are needed to confirm the rise of mitochondrial Ca^2+^ load. Moreover, the ETC is partially un-coupled and H_2_O_2_ production is increased in association with mitochondria fragmentation in prediabetic hearts. In AC3-I mice, in which myocardial CaMKII is inhibited, these events are prevented. The overexpression of the tethering and fission proteins described are CaMKII-dependent. Since CaMKII inhibition avoids SR-Ca^2+^ leak and the following steps described as the underlying signaling (although we used WT mice for the forward experiments after established increased SCaRE in FRD mice), we propose that enhanced Ca^2+^ at the microdomains is the pivotal event in the pathogenesis of prediabetic hearts. However, further investigations across the time are required to asseverate that this molecular signaling in the prediabetic state is the basis of diabetic cardiomyopathy.

## Methods

Full descriptions of methods used are available in the “Supplementary materials” section.

### Ethical approval

All experiments were performed in accordance to the Guide for Care and Use of Laboratory Animal and approved by the Ethics committee of the Faculty of Medicine, La Plata, Argentina Institutional Animal Care and Use Committee (CICUAL no. P03-02-2017). All experiments were performed following the recommendation of ARRIVE guidelines.

### Animal model and protocols

Male transgenic mice with cardiomyocyte-delimited transgenic expression of a CaMKII inhibitor peptide, AC3-I^70^, generously supplied by Dr. Mark Anderson (Baltimore, MD, USA), was reproduced and genotyped in our laboratory. As control animals, we used either C57bl/6 or inactive scrambled control peptide (AC3-C) mice. The AC3-C mice were used only for SCaRE measurements since the AC3 mice line co-express the GFP protein which interferes with the Ca^2+^ signal. The rest of the experiments were performed with C57bl/6 (WT) mice as control. Animals were divided into two groups: control diet group (CD), fed with a standard commercial diet, and fructose-rich diet group (FRD), which received the same diet plus 10% (w/v) fructose in the drinking water for 3 weeks. The FRD is an already validated model of prediabetes in which animals develop impaired glucose tolerance, hyperinsulinemia, and insulin resistance^19–21^.

After treatment, animals were sacrificed by cervical dislocation and the heart was immediately excised by central thoracotomy. At that moment, hearts were assigned for biochemical studies, transmission electron microscopy (TEM), [^3^H]Ryanodine binding assays, myocyte isolation, or mitochondria isolation, fully described in Supplementary Material Section.

### Cardiomyocyte isolation

Cardiomyocytes were isolated by enzymatic digestion as previously described^71^.

### Confocal microscopy

Confocal images of Ca^2+^ sparks, waves, and spontaneous contractile activity were captured in line scan mode.

### [^3^H]Ryanodine binding assays

Binding assays were carried out following a modified version of a protocol previously described^72^.

### Western blotting

Hearts were freeze-clamped, pulverized, and processed as previously described^21,73^. The analysis of the immunoblots was performed by Fiji program. For sake of clarity, representative blots were selected to be shown in the main figures, and the full-length gels are presented in Supplementary Figure S3.

### Mouse heart isolated mitochondria

Animals were anesthetized, and hearts were immediately excised. Heart mitochondrial purified fractions were obtained as described earlier^74^ by differential centrifugation. The isolated mitochondria were used in fluorescence spectroscopy using a microplate reader (Varioskan® LUX, Thermo Scientific, MA, USA) to measure different mitochondrial parameters. For ATP production rate^75^ we used the luciferin/luciferase assay, for H_2_O_2_ production rate Amplex Red were used^76^, and for calcium retention capacity (CRC)^77^ we performed sequential addition of 10 μM CaCl_2_ until the mitochondria reached a CaCl_2_ saturation point. Mitochondrial O_2_ consumption was measured with a Clark-type O_2_ electrode for high-resolution respirometry^74^.

### Transmission electron microscopy

Strips from the middle of the left ventricle wall, avoiding the apex and the base area, were cut in 1 mm^3^ samples. Tissue samples were fixed in 2% glutaraldehyde at 4 °C and processed as previously described^22^. TEM images were used to measure mitochondria roundness, diameter, SR-mitochondria distance, and mitochondria density^22^.

### Statistics

The statistical analyses were performed using *GraphPad Prism 8.0.1* program. The assumption of normality was examined using the Shapiro-Wilk test. When *P* > 0.05 distribution of data was considered normal. Continuous variables were expressed as mean ± SEM and were evaluated with either an unpaired Student’s t-test, or a Mann Whitney test for not normal populations.

A *P* < 0.05 value was used to indicate statistical significance. The exact *P* value was indicated in the figures. The number of experiments and the corresponding number of animals, between parenthesis, were indicated either in the figures or its legends.

### Consent for publication

All authors gave their consent for all data publication.

## Supplementary Information

Supplementary Information.

## Data Availability

The datasets generated and/or analyzed during the current study are available on reasonable request.
