# Supplementary material for: CaMKII activation in early diabetic hearts induces altered sarcoplasmic reticulum-mitochondria signaling
Source: Sci Rep. 2021 Oct 8;11:20025. doi: 10.1038/s41598-021-99118-x (PMC8501049; doi:10.1038/s41598-021-99118-x)
Supplement: Supplementary file 1 — Supplementary Information. [file 41598_2021_99118_MOESM1_ESM.pdf]

CaMKII activation in early diabetic hearts induces altered sarcoplasmic reticulum-mitochondria signaling

By

Federico Marilen, Zavala Maite, Vico Tamara, López Sofía, Portiansky Enrique, Alvarez Silvia, Villa Abrille M Celeste, Palomeque Julieta

Supplementary material

Methods

Cardiomyocyte isolation

Cardiomyocytes were isolated by enzymatic digestion as previously described[1]. Briefly, hearts were attached to a cannula via the aorta, mounted and retrogradely perfused in a Langendorff system, at 37 °C and constant coronary flow. For perfusion, the following solution was used: 146.2 mM NaCl, 4.7 mM KCl, 10.0 mM HEPES, 0.35 mM NaH_2PO_4, 1.05 mM $MgSO_4$, 11.1 mM glucose and 0.1 mM EGTA (pH adjusted to 7.4 with NaOH). The solution was continuously bubbled with 100% O_2. Hearts were then perfused with the above described solution supplemented with collagenase (270 U/ml), 1 % BSA, and 50 μM $CaCl_2$. Perfusion continued until hearts became flaccid (15–20 min). Following this perfusion procedure, cardiomyocytes were manually dissociated. Ventricular myocytes were then slowly recalcified and kept at room temperature in a solution containing 1 mM Ca^{2+}. Only rod-shaped cardiomyocytes with clear and distinct striations were used. Experiments were performed at room temperature (22-23 ˚C) and carried out in the above described solution in the presence of 2 mM Ca^{2+} (pH 7.4; equilibrated with 100% O_2).

Confocal microscopy

Confocal images of Ca^{2+} sparks, waves, and spontaneous contractile activity were captured in line scan mode. Isolated cardiomyocytes were loaded with 10 μM Rhod-2 AM for 30 min at room temperature and then excited with a 525 nm argon laser. Fluorescence was collected above 580 nm[2]. Each image consisted of 512 line-scans obtained at 4 ms intervals in a Confocal Zeiss LSM 410.

Data were visualized and Ca^{2+} sparks were measured using Fiji. Sparks, waves and spontaneous contraction of quiescent cells were obtained. Ca^{2+} sparks release events (SCaRE) frequency was expressed as % SCaRE/μm.sec.

[^{3}H]Ryanodine binding assays

Binding assays were carried out following a modified version of a protocol previously described[3]. Binding mixtures were prepared to contain 50 μg of protein from heart

homogenates, 200 mM KCl, 20 mM Na-HEPES (pH 7.4), 6.5 nM [^{3}H]Ryanodine (Perkin Elmer, Boston, MA, USA) and enough $CaCl_2$ to set free Ca^{2+} concentration between 10 nM and 100 μM. EGTA (1 mM) was used to buffer Ca^{2+}. The Ca^{2+}/EGTA ratio for these solutions was determined using MaxChelator (https://somapp.ucdmc.ucdavis.edu/pharmacology/bers/maxchelator/). The binding reactions were incubated for 2 h in a thermostatic bath with a shaker at 37 °C, filtered through Whatman GF/B filters presoaked with bi-distilled water and then washed three times with 5 ml of bi-distilled water in a Brandel M24-R Harvester. Non-specific binding was determined in the presence of 20 μM unlabelled Ryanodine (MP Biomedicals). [^{3}H]Ryanodine ([^{3}H]Ry) binding was determined by liquid scintillation. Hill's equation was used to determine the maximum [^{3}H]Ry binding (B_{max}) in Origin 9 (Origin Lab, Northampton, MA, USA).

Western blotting

Hearts were freeze-clamped, pulverized, and processed as previously described[4]. Briefly, left ventricle was homogenized in four volumes of lysis buffer (20 mM sodium glycerolphosphate, 20 mM NaF, 1 mM EGTA, 2 mM EDTA, 0.2 mM Na_2VO_4, 2 mM dithiothreitol, 10 mM benzamide, 1 mM phenylmethylsulfonyl fluoride, 0.001 mM pepstatin, 1 % Igepal, 0.01 % Triton and 0.048 mg/mL leupeptin). Protein was measured by the Bradford method using BSA as standard. Lysates (60 μg of total protein) were separated per gel line in 10% SDS polyacrylamide gel[5] and transferred to polyvinylidene difluoride (PVDF) membranes. The membrane obtained were bloked in 5% milk solution for 1h. Blots were incubated overnight with the following primary antibodies: Mfn2 (1:1000, Santa Cruz Biotechnology, Inc., Santa Cruz, CA, USA), Grp75 (1:500, Santa Cruz Biotechnology, Inc., Santa Cruz, CA, USA), VDAC (1:1000, SIGMA Aldrich, USA), Opa1 (1:1000, Santa Cruz Biotechnology, Inc., Santa Cruz, CA, USA), and Drp1 (1:1000, Santa Cruz Biotechnology, Inc., Santa Cruz, CA, USA). GAPDH or Na^+/K^+ ATPase signals were used to normalize the amount of each protein. Secondary antibodies were used as appropriate, goat anti-mouse horseradish peroxidase (HRP) (1:15000, Santa Cruz Biotechnology, Inc., Santa Cruz, CA, USA) or goat anti-rabbit HRP (1:15000, Santa Cruz Biotechnology, Inc., Santa Cruz, CA, USA). The signals were recorded in a Chemidoc BioRad equipment. The followed analysis was done using Fiji program.

Mitochondria isolation

Animals were anesthetized, and hearts were immediately excised. Heart mitochondrial purified fractions were obtained as described earlier[6] by differential centrifugation in a refrigerated centrifuge (Sorvall RC5C, Buckinghamshire, England). Briefly, left ventricles were dissected, washed, and minced in ice-cold STE buffer (250 mM sucrose, 5 mM Tris-HCl, and 2 mM EGTA, pH 7.4). Then, tissues were digested in 2.5 mL of STE buffer supplemented with 2.5 UI/mL type XXIV bacterial proteinase plus 0.5% p/v BSA, 5 mM $MgCl_2$, and 1 mM ATP for 4 min at 4 °C. After digestion, 2.5 mL of STE buffer was added and the preparation was homogenized with a Potter Elvejhem glass homogenizer. Homogenates were then centrifuged at 8000 *g* for 10 min to discard the excess of proteinase. Pellets were resuspended in 4 mL STE buffer and centrifuged at 700 *g* for 10 min to discard nuclei and cell debris. The supernatant was then centrifuged at 8000 *g* for 10 min to isolate the mitochondrial fraction. Finally, the pellet was washed and resuspended in the same isolation buffer obtaining a final protein concentration of 10 mg/mL. The whole procedure was carried out at 0 – 4°C. The isolated fraction consisted of mitochondria able to carry out oxidative phosphorylation. Protein concentration was measured by the Lowry assay using BSA as a standard[7].

Mitochondrial O_2 consumption

Mitochondrial O_2 consumption was measured using a Clark-type O_2 electrode for high-resolution respirometry (Hansatech Oxygraph, Hansatech Instruments Ltd., Norfolk, England). Freshly isolated mitochondria (0.4 - 0.6 mg/mL) were incubated in a respiration medium at 30 °C (120 mM KCl, 5 mM KH_2PO_4, 1 mM EGTA, 3 mM HEPES, 1 mg/mL BSA, pH 7.2). Resting respiration state (state 4) was measured in the presence of 2 mM malate and 5 mM glutamate as substrates, and then followed by the addition of 1 mM AD; thus, an active respiration state (state 3) was obtained. Results were expressed as atom-oxygen nanograms per min per milligrams of protein (ng-atoms O_2/min.mg protein). Respiratory control ratio (RCR) was calculated as the ratio between state 3 and state 4 respiration rates[6,8].

Mitochondrial ATP production rate

ATP production was measured in freshly isolated mitochondria using the luciferin/luciferase assay in a microplate reader (Varioskan® LUX, Thermo Scientific, MA, USA). Freshly isolated cardiac mitochondria (1 mg/mL) were incubated in a reaction medium at 30 °C (150 mM KCl, 25 mM Tris-HCl, 2 mM EDTA, 0.1% BSA, 10 mM K_2HPO_4/KH_2PO_4, 0.1 mM $MgCl_2$, 0.8 mM luciferin, 20 μg/mL luciferase, pH 7.4). To determine the ATP production rate, 3 mM malate, 1.25 mM glutamate, and 125 µM ADP, were added to the reaction medium. A calibration curve was performed using ATP as a standard. Results were expressed as % of the CD ATP production rate[9].

Mitochondrial hydrogen peroxide (H_2O_2) production rate

To study ROS generation from freshly isolated mitochondria, H_2O_2 production was measured by fluorescence spectroscopy using the Amplex Red/HRP system. Freshly isolated mitochondria were incubated in a reaction medium (125 mM sucrose, 65 mM KCl, 10 mM HEPES, 2 mM KH_2PO_4, 2 mM $MgCl_2$, 0.01% BSA, pH 7.2) in the presence of 25 μM Amplex Red, 0.5 U/mL HRP, and 2 mM malate and 5 mM glutamate as substrates. Resorufin fluorescence intensity (the product of Amplex Red oxidation by H_2O_2/HRP) was measured in a microplate reader (Varioskan® LUX, Thermo Scientific, MA, USA) at 563 nm and collected at 587 nm. A calibration curve was obtained using H_2O_2 as standard. Results were expressed as % of the control H_2O_2 production rate[10].

Mitochondrial Ca^{2+} retention capacity

Calcium retention capacity (CRC) assay was performed in a temperature-controlled in a microplate reader (Varioskan® LUX, Thermo Scientific, MA, USA. 1 mg of freshly isolated mitochondria were pre-incubated at 37°C in a medium containing: 120 mM KCl, 20 mM MOPS, 10 mM Tris-HCl and 5 mM KH_2PO_4, pH 7.4, and loaded with 0.5 µmol/L Calcium green-5N salt (Invitrogen, Carlsbad, CA, USA). Mitochondria were then pulsed with sequential additions of 10 μM $CaCl_2$ until the mitochondria reached a $CaCl_2$ saturation point and could no longer take up more Ca^{2+}. The fluorescence excited at 506 nm and emitted at 532 nm was recorded. CRC was expressed in nmol of Ca^{2+}/mg mitochondrial protein, where

we count the pulses and calculate de total Ca^{2+} uptake in nM (number of peaks x 10 μM) and normalize by milligrams of mitochondria protein used[11].

Transmission electron microscopy

Animals of each strain and treatments were anesthetized and hearts were immediately excised. The left ventricle was dissected from the right and the atria. Then, strips from the middle of the left ventricle wall, avoiding the apex and the base area, were cut in 1 mm^3 samples. Tissue samples were fixed in 2% glutaraldehyde at 4°C. Each set was washed in phosphate buffer (168 mM Na_2HPO_4, 49 mM NaH_2PO_4), postfixed in 1% OsO_4 for 1 h at 4 °C, dehydrated in a graded series of alcohol from 50° to acetone and embedded in low-viscosity epoxy resin[12] as described previously. Polymerization was performed for 24 h at 60 °C. Ultrathin sections with interference color grey were cut with an ultramicrotome (Ultracut R; Leica EM UC7), mounted on grids, and stained with 1% uranyl acetate and lead citrate [13]. Grids were examined by TEM (JEOL JEM 1200; Zeiss) using a digital camera (Erlangshen ES1000).

Morphometry analysis. TEM images were used to measure different morphometric parameters as previously described[14]. Mitochondria roundness and diameter were measured on 12000x magnification images. Roundness reports how round is an object (mitochondria) as compared to a circle. Circular objects will have a roundness = 1; other shapes will have a roundness different to 1. It is determined by the following formula: $Roundness\ Index = \frac{Pm^2}{(4 \times \pi \times A)}$, where Pm is the perimeter and A is the area. SR-mitochondria distance was measured on 80000x magnification images. For the latter, thresholded images were skeletonized to establish the centerline of the SR membrane and of the OMM. At least ten perpendicular lines were then traced between both skeletonized lines and their average size was represented as the distance between both organelles. Moreover, the space between organelles was colored for better discrimination of the microdomains.

Mitochondrial density quantification. Samples were fixed as described before to obtain the TEM images. Mitochondria density quantification was carried out on at least nine 12000x and/or 30000x magnification images. Quantification was normalized per μm^2 of the image to express density as number/μm^2 (nº/μm^2).

Mitochondrial holes quantification. Lesser electrodense areas (holes area) present in the mitochondria were quantified in TEM photographs in 30000x and 12000x magnification. Either the holes or total mitochondrial areas were skeletonized using the program ImageProPlus. The holes areas were normalized by the total mitochondrial area.

References

1. Palomeque, J. *et al.* Angiotensin II–Induced Oxidative Stress Resets the Ca $^{2+}$ Dependence of Ca $^{2+}$ –Calmodulin Protein Kinase II and Promotes a Death Pathway Conserved Across Different Species. *Circulation Research* **105**, 1204–1212 (2009).
2. MacGowan, G. A. *et al.* Rhod-2 based measurements of intracellular calcium in the perfused mouse heart: Cellular and subcellular localization and response to positive inotropy. *J. Biomed. Opt.* **6**, 23 (2001).
3. Helms Adam S. *et al.* Genotype-Dependent and -Independent Calcium Signaling Dysregulation in Human Hypertrophic Cardiomyopathy. *Circulation* **134**, 1738–1748 (2016).
4. Sommese, L. *et al.* Ryanodine receptor phosphorylation by CaMKII promotes spontaneous Ca(2+) release events in a rodent model of early stage diabetes: The arrhythmogenic substrate. *International Journal of Cardiology* **202**, 394–406 (2016).
5. Mundiña-Weilenmann, C., Vittone, L., Ortale, M., de Cingolani, G. C. & Mattiazzi, A. Immunodetection of Phosphorylation Sites Gives New Insights into the Mechanisms Underlying Phospholamban Phosphorylation in the Intact Heart. *J. Biol. Chem.* **271**, 33561–33567 (1996).
6. Vico, T. A. *et al.* Mitochondrial bioenergetics links inflammation and cardiac contractility in endotoxemia. *Basic Res Cardiol* **114**, 38 (2019).
7. Lowry, G. G. *Lowrys' Handbook of Right-to-Know and Emergency Planning, Sara*. (CRC Press, 1988).
8. Boveris, A., Costa, L. E., Cadenas, E. & Poderoso, J. J. [20] Regulation of mitochondrial respiration by adenosine diphosphate, oxygen, and nitric oxide. in *Methods in Enzymology* vol. 301 188–198 (Academic Press, 1999).

9. Vives-Bauza, C., Yang, L. & Manfredi, G. Assay of Mitochondrial ATP Synthesis in Animal Cells and Tissues. in *Methods in Cell Biology* vol. 80 155–171 (Academic Press, 2007).

10. Chen, Q., Vazquez, E. J., Moghaddas, S., Hoppel, C. L. & Lesnefsky, E. J. Production of Reactive Oxygen Species by Mitochondria: CENTRAL ROLE OF COMPLEX III. *J. Biol. Chem.* **278**, 36027–36031 (2003).

11. Li, W., Zhang, C. & Sun, X. Mitochondrial Ca^{2+} Retention Capacity Assay and Ca^{2+}-triggered Mitochondrial Swelling Assay. *JoVE* 56236 (2018) doi:10.3791/56236.

12. Spurr, A. R. A low-viscosity epoxy resin embedding medium for electron microscopy. *Journal of Ultrastructure Research* **26**, 31–43 (1969).

13. Reynolds, E. S. THE USE OF LEAD CITRATE AT HIGH pH AS AN ELECTRON-OPAQUE STAIN IN ELECTRON MICROSCOPY. *J Cell Biol* **17**, 208–212 (1963).

14. Federico, M. *et al.* Calcium-calmodulin-dependent protein kinase mediates the intracellular signalling pathways of cardiac apoptosis in mice with impaired glucose tolerance. *The Journal of Physiology* **595**, 4089–4108 (2017).

Supplementary Figures

Figure S1. Disarrangement tissue in prediabetic heart mice.

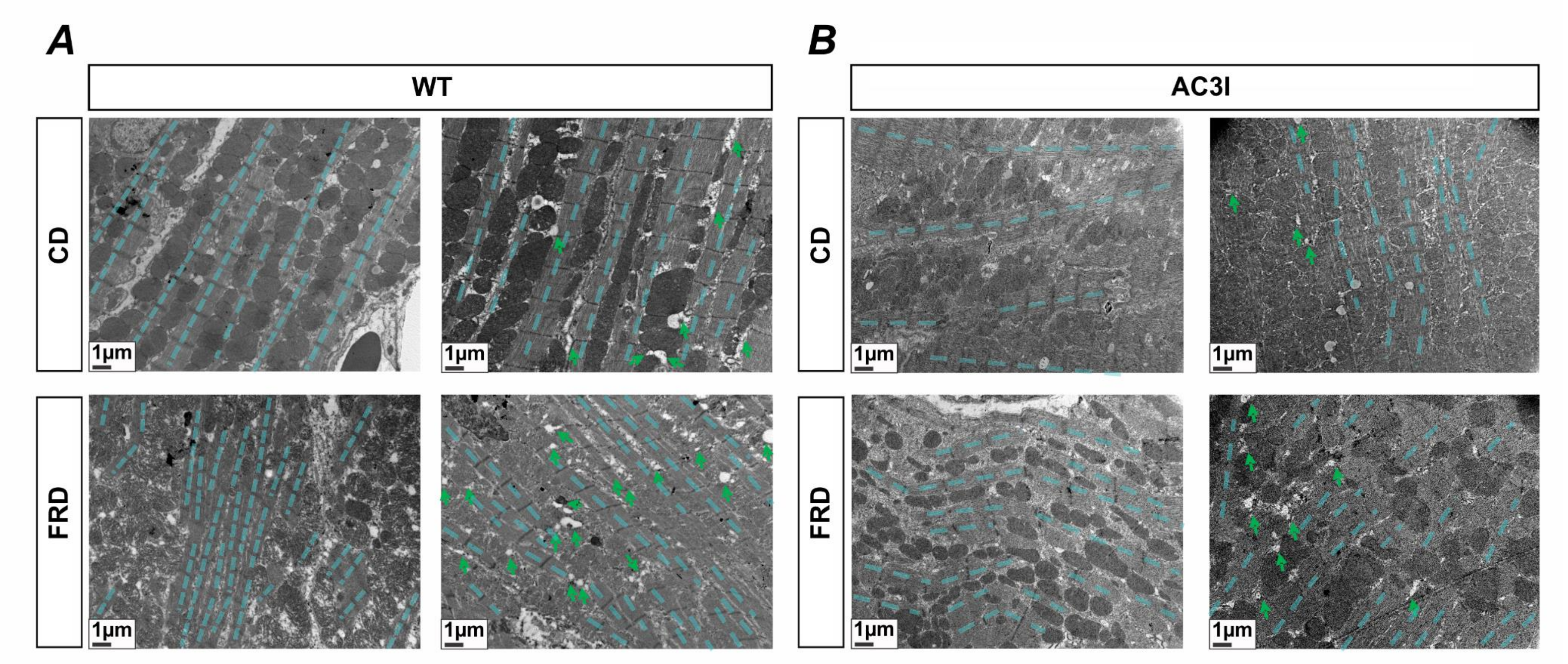

Representatives transmission electron microscopy (TEM) images of different specimens of heart tissue from ***A)*** CD and FRD WT and ***B)*** CD and FRD AC3-I, at 12000x magnification. Dotted lines show the arrangement of myofibrils between aligned mitochondria while arrows point to vacuoles in the tissue. ***A)*** FRD WT myofibrils and mitochondria do not follow the same arrangement pattern as shown by CD WT sections. Similar patterns were observed in all the samples analyzed. ***B)*** In FRD AC3-I tissue the arrangement of myofibrils is partially conserved between the aligned mitochondria. Also, the number of vacuoles that appear in cardiac tissue is notoriously increased in FRD WT hearts but not in FRD AC3-I as compared with their respective control samples. The scale bar for all figures = 1 μm.

Figure S2. Full-length western blots from WT heart mice.

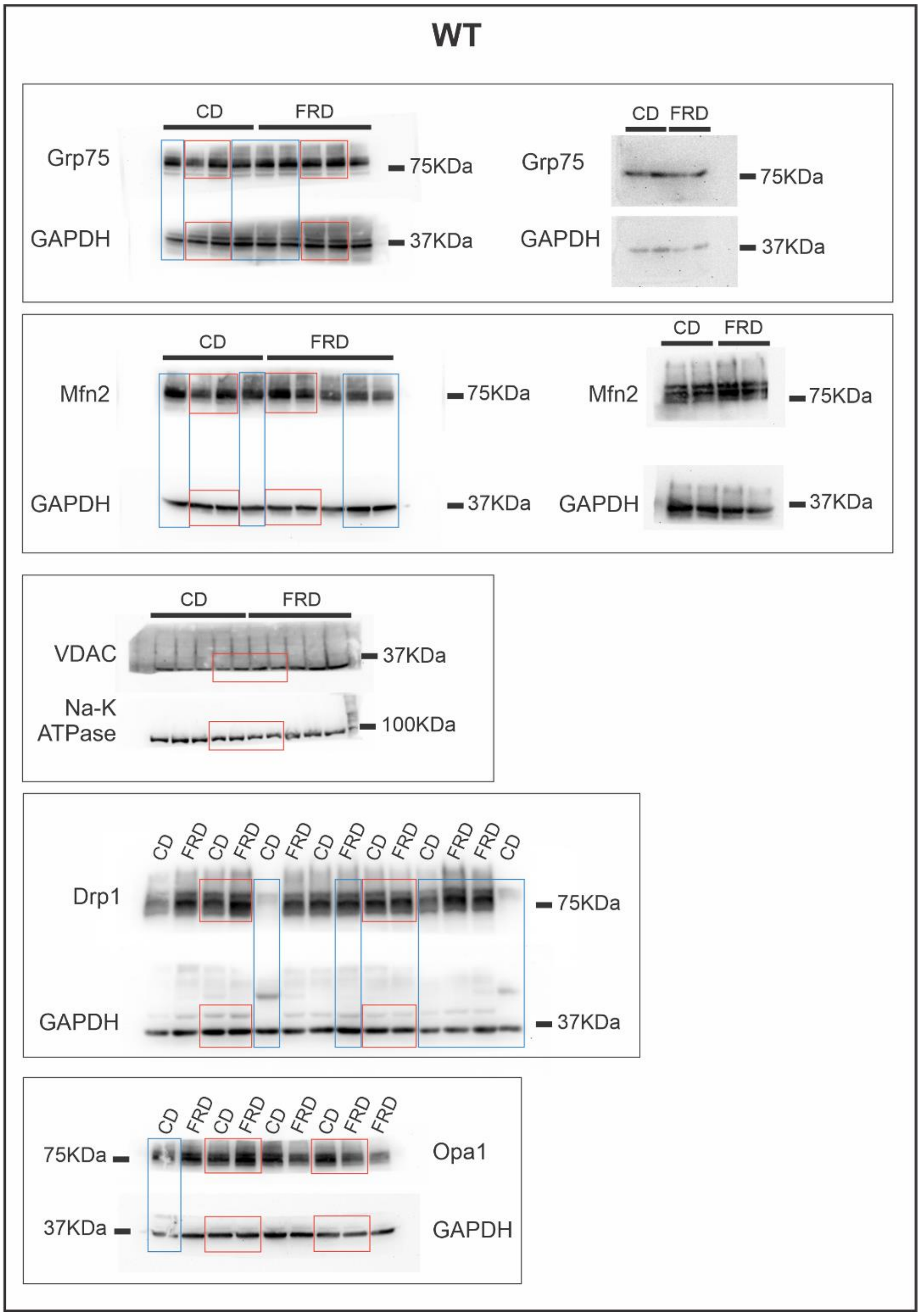

Full-length western blot of figures 2B and 5. The red squares, delimitate the representative blots selected to be shown in the figure of the main text. The blue squares, delimited blots of samples that were disregarded for visible experimental reasons (broken gel, tail effect, bubbles, smeared bands, repeated samples, etc). The blots that are not delimitated were used for average data. Full gels are not available since once running and transferred, the membranes were cut according to the molecular weight of the primary antibody to search.

Figure S3. Full-length western blots from AC3-I heart mice.

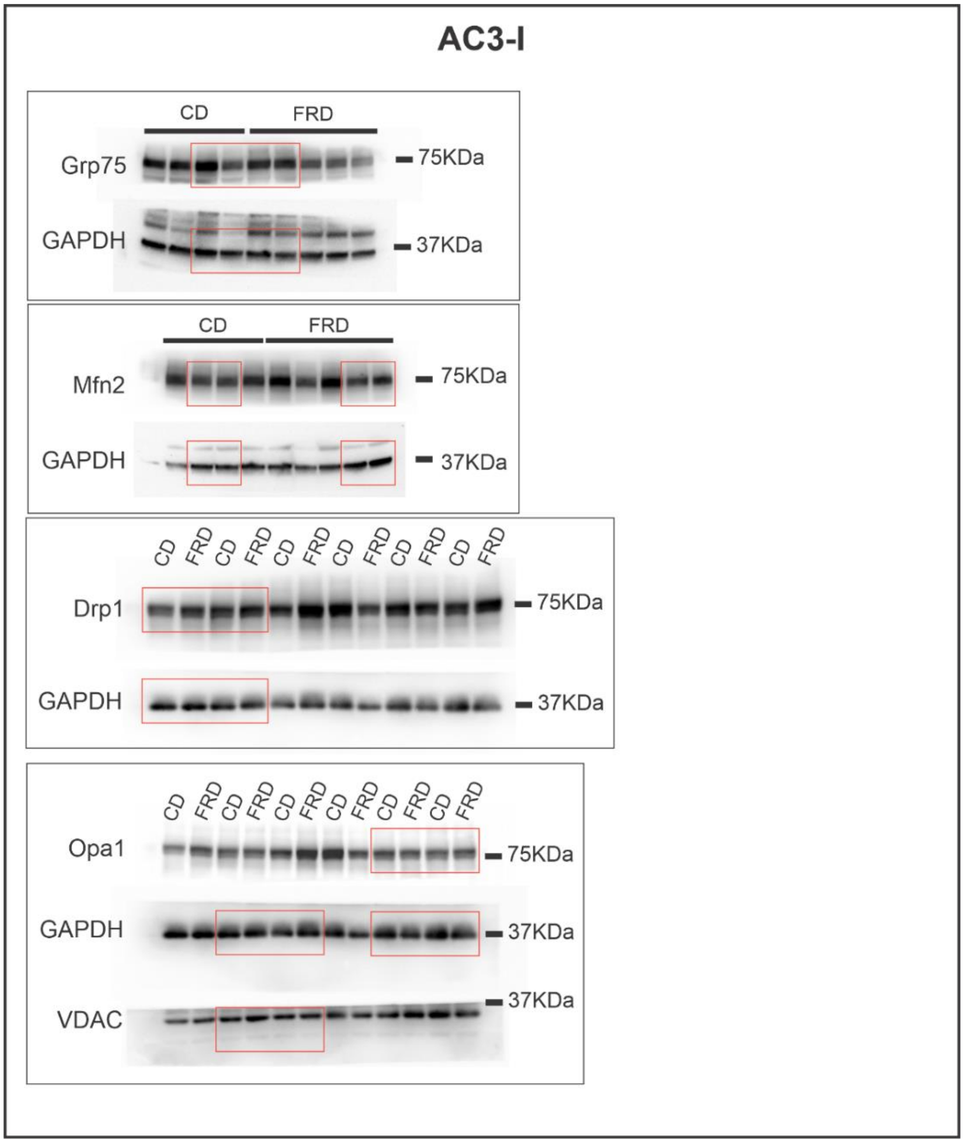

Full-length western blot of figures 6B and 9. The red squares delimitate the representative blots selected to be shown in the main figure.
